# Repellency of forty‐one aromatic plant species to the Asian citrus psyllid, vector of the bacterium associated with huanglongbing

**DOI:** 10.1002/ece3.6876

**Published:** 2020-11-03

**Authors:** Zhaogui Yan, Qun Zhang, Nan Zhang, Wan Li, Cuiying Chang, Yan Xiang, Changxiu Xia, Tengyu Jiang, Wei He, Jie Luo, Yongrong Xu

**Affiliations:** ^1^ College of Horticulture and Forestry Sciences/Hubei Engineering Technology Research Center for Forestry Information Huazhong Agricultural University Wuhan China; ^2^ School of Environmental & Rural Science The University of New England Armidale NSW Australia; ^3^ Ganzhou Citrus Science Research Institute Ganzhou China; ^4^ Zhongnan Institute of Survey Ministry of Forestry and Grass Changsha China

**Keywords:** biological control, citrus greening disease, *Diaphorina citri*, insect vector, plant repellency, volatile

## Abstract

Huanglongbing (HLB) is the most devastating citrus disease worldwide. The organism associated with the disease is spread by an insect vector, *Diaphorina citri*, commonly known as Asian citrus psyllid (ACP). Current management of HLB relies either on physical removal of the infected plants or on chemical control of ACP. Both methods are costly and not overly effective. In addition, public concerns regarding insecticide residues in fruit have greatly increased in recent years. It has been hypothesized that plant volatiles could act as repellents to ACP, thus reduce the incidence of HLB. To test this hypothesis, the repellency of fresh tissues of 41 aromatic plant species to ACP was investigated. The repellency of individual species was determined using a Y‐tube olfactometer. Our results showed that volatiles of five plant species were highly effective in repelling ACP with repellency as much as 76%. Among these, the tree species, *Camptotheca acuminate*, *a*nd the two shrubs, *Lantana camara* and *Mimosa bimucronata*, could potentially be planted as a landscape barrier. The two herbs, *Capsicum annuum* and *Gynura bicolor*, could potentially be used as interplantings in orchards. This is the first time that the repellency of fresh tissues from a diverse range of plant species to ACP has been determined. Although further field evaluation of various interplanting regimes and landscape barriers are needed to assess their effectiveness, our results showed that these aromatic species, being highly repellent to ACP, offer great potential as more cost‐effective and environmentally sustainable alternatives to the current methods of managing HLB.

## Graphical abstract

The repellency of 41 species to *Diaphorina citri* was determined. Volatiles of five plant species were highly effective in repelling *Diaphorina citri*. Repellency was as high as 76%. These five species offer potential for managing citrus Huanglongbing.

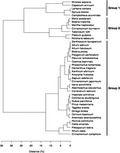

## 1 INTRODUCTION

Huanglongbing (HLB) or greening disease is the most devastating citrus disease worldwide and is considered a major threat to global citrus industry (Bove, 2006). The disease is prevalent in all major citrus growing regions, including Africa, Asia, and North and South America (Bove, 2006; Yan et al., 2015), and causes considerable economic losses. In China, the disease is prevalent in 11 of the 19 citrus growing provinces; in Guangdong alone, the disease causes annual economic loss of over 10 billion CNY (or 1.5 billion USD) (Qin, 2018). In Florida, United States, where HLB was first recorded in 2003 and has since been found in 80% of commercial orchards; it has caused a 71% decline in fruit production (170 million boxes of about 41 kg/box) in the 2007–08 season to 45 million boxes in the 2017–18 season (US Department of Agriculture, 2018).

The disease is associated with three phloem α‐proteobacterias, *Candidatus* Liberibacter asiaticus, *Ca*. Liberibacter americanus and *Ca*. Liberibacter africanus. After infection, the bacterium lives in the phloem of the infected plants. Infected plants can remain symptom‐free for up to 5 years (Gottwald, 2010). The Asian strain of the disease, prevalent in China and Southeast Asia, is caused by *Ca*. L. asiaticus and is spread by an insect vector, the Asian citrus psyllid (ACP), *Diaphorina citri* (Gottwald et al., 2014; Halbert & Manjunath, 2004; Yan et al., 2015). The disease affects fruit production by causing premature fruit drop and producing fruit with low economic value (Bassanezi et al., 2009; Plotto et al., 2010) or causing whole plant death in extreme cases (Zheng et al., 2018).

Currently, there is no adequate treatment for infected plants. Management of the disease relies either on physical removal or pruning of the infected plants, or on chemical control of the insect vector (Bove, 2006; Wang, 2019). Pruning of infected parts of plants is costly and not overly effective given the long latent period of the disease (Bassanezi et al., 2013; Rouse et al., 2017) and is considered a non‐viable approach for HLB management (Rouse et al., 2017; Vashisth & Livingston, 2019) Using insecticides to control ACP is presently the primary option for HLB management. However, increase in the resistance of ACP to various insecticides has reduced their effectiveness (Hall et al., 2013; Ichinose et al., 2010; Naeem et al., 2016). In addition, insecticides can have adverse effects on non‐target species (Beloti et al., 2015) and increasingly becoming a health hazardous both to the citrus workers and to the general public (Beloti et al., 2015; Wang, 2019). Plant volatiles capable of repelling ACP have been suggested as a cost‐effective and environmentally friendly alternative to current methods of HLB management.

Plant volatiles have been used in pest management. For instance, plant volatiles have been used to repel insects, such as mosquitos, the vector of the pathogen of malaria with various levels of success (Diaz, 2016; Hill et al., 2007; Karunamoorthi et al., 2009; Moore et al., 2002) and stored‐product pests (*Tribolium castaneum* and *Liposcelis bostrychophila*) (Yang et al., 2015). In horticulture, volatiles from a garlic‐pepper combination were effective in controlling Guatemalan potato moth (*Tecia solanivora*) in potato crops (Jimenez & Poveda, 2009). Aromatic species, *Saturela hortensis* and *Agerarum houstonianum*, interplanted with pears (*Pyrus pyrifolia*) halved the abundance of scarab beetles (*Serica orientalis*) (Tang et al., 2013). Similarly, volatiles have been used for ACP control. In laboratory and field studies, volatiles from guava (*Psidium guajava*) were effective in repelling ACP and reducing the incidence of HLB (Barman et al., 2016; Gottwald et al., 2014; Zaka et al., 2010). It is therefore hypothesized that using volatiles from aromatic plants in HLB management could be a cheaper, more effective, and more environmentally friendly alternative to current methods, since these volatiles could repel ACP.

To test the hypothesis that volatiles of aromatic plants can effectively repel citrus psyllids, the repellency of volatiles of 41 aromatic plant species to ACP in Ganzhou, Jiangxi Province, China were investigated. Most of the selected plant species occur locally in Ganzhou. Our objective was to identify plants with volatiles that are effective in repelling ACP in order to improve the control of HLB in citrus.

Ganzhou is one of the major citrus growing regions of China. HLB is prevalent in the region and has caused significant economic loss. Finding a cost‐effective, and environmentally friendly solution to HLB is imperative. This study formed part of the national program for scientific management of HLB in the Chinese citrus industry.

## 2 MATERIALS AND METHODS

### 2.1 Adult psyllids (*Diaphorina citri*)

Live adult psyllids were collected from the orchards of Ganzhou Citrus Research Centre, Jiangxi Province, China. These psyllids were collected on the day of the experiment to ensure that they were active and vigorous during the experiment. Before the experiment, the psyllids were deprived of food for 2 hr to ensure that they were actively seeking food. We did not examine individual psyllids for bacterial infection. As these psyllids were collected in orchards of HLB prevalent areas, it is likely that some of the psyllids were infected. While we know little about the potential effect of bacterial infection on psyllid behavior in response to volatiles, this effect would have been minimized by (a) the large number of psyllids (40 per plant species) used per experiment and (b) similar percentage of bacterial infection of the 40 psyllids as these psyllids were randomly selected from freshly captured pools.

### 2.2 Plant species selection

Forty‐one species of plants were selected for evaluation (Table 1). This selection was based on (a) moderate to high concentrations of foliar volatiles, and (b) occurrence in citrus growing regions of China. Plant materials were collected locally where possible. Many of the selected species were obtained as nursery stock in local markets. These plants were kept in a greenhouse at the Ganzhou Citrus Research Centre until used. The commonly grown citrus, *Citrus reticulata* cv. Murcott, was used as the control plant.

**TABLE 1:** List of plant species tested for repellency to Asian citrus psyllids (*Diaphorina citri*)

Test plant species	Family	Plant type	Source^a^
*Abelmoschus esculentus*	Malvaceae	Herb	Cultivated
*Allium cepa*	Liliaceae	Herb	Cultivated
*Allium fistulosum*	Liliaceae	Herb	Cultivated
*Allium sativum*	Liliaceae	Herb	Cultivated
*Amorpha fruticosa*	Leguminosae	Shrub	Wild
*Artemisia lavandulaefolia*	Asteraceae	Herb	Wild
*Bidens pilosa*	Asteraceae	Herb	Wild
*Bidens tripartita*	Asteraceae	Herb	Wild
*Camptotheca acuminata*	Nyssaceae	Tree	Wild
*Capsicum annuum*	Solanaceae	Herb	Cultivated
*Celtis sinensis*	Ulmuceae	Tree	Wild
*Cinnamomum burmanni*	Lauraceae	Tree	Cultivated
*Cinnamomum camphora*	Lauraceae	Tree	Wild
*Cinnamomum japonicum*	Lauraceae	Tree	Wild
*Corchorus acutangulus*	Tiliaceae	Herb	Wild
*Coriandrum sativum*	Apiaceae	Herb	Cultivated
*Cosmos bipinnatus*	Asteraceae	Herb	Wild
*Ginkgo biloba*	Ginkgoaceae	Tree	Wild
*Gynura bicolor*	Asteraceae	Herb	Cultivated
*Imperata cylindrica*	Gramineae	Herb	Wild
*Crepidiastrum sonchifolium*	Asteraceae	Herb	Wild
*Lantana camara*	Verbenaceae	Shrub	Wild
*Melia azedarach*	Meliaceae	Tree	Wild
*Mentha haplocalyx*	Labiatae	Herb	Cultivated
*Mimosa bimucronata*	Leguminosae	Shrub	Wild
*Nicotiana tabacum*	Solanaceae	Herb	Wild
*Ocimum basilicum*	Labiatae	Herb	Cultivated
*Osmanthus fragrans*	Oleaceae	Tree	Wild
*Paliurus ramosissimus*	Rhamnaceae	Shrub	Wild
*Pinus massoniana*	Pinaceae	Tree	Wild
*Pittosporum tobira*	Pittosporaceae	Shrub	Wild
*Polygonum perfoliatum*	Polygonaceae	Herb	Wild
*Psidium guajava*	Myrtaceae	Tree	Cultivated
*Ricinus communis*	Euphorbiaceae	Herb	Cultivated
*Rhodomyrtus tomentosa*	Myrtaceae	Shrub	Cultivated
*Rubus parvifolius*	Rosaceae	Shrub	Wild
*Sapium sebiferum*	Euphorbiaceae	Tree	Wild
*Tagetes erecta*	Asteraceae	Herb	Cultivated
*Tuberosum rottl*	Liliaceae	Herb	Cultivated
*Xanthium sibiricum*	Asteraceae	Herb	Wild
*Zanthoxylum bungeanum*	Rutaceae	Shrub	Cultivated

^a^ Cultivated plants are those sourced from local nurseries; wild plants are those collected in the field.

### 2.3 Repellency assessment

**FIGURE 1:**
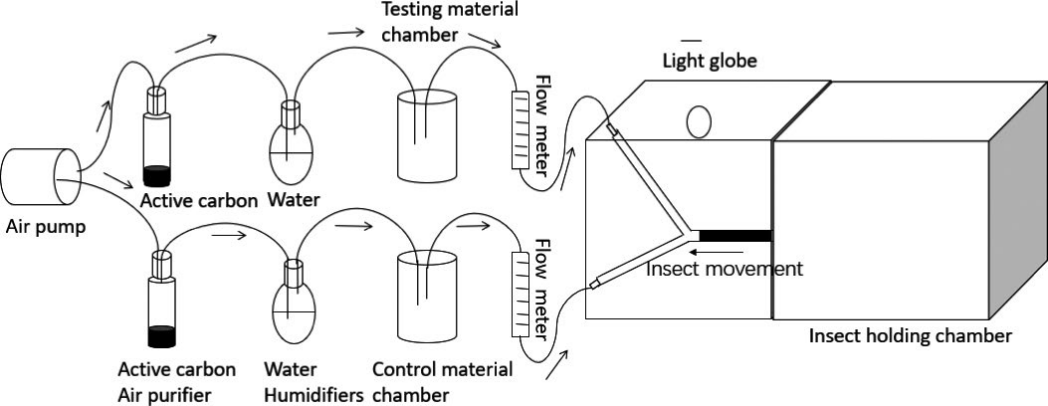
A schematic view of Y‐tube olfactometer. From left to right: air pump, active carbon air‐purifiers, humidifiers, test and control chambers, airflow meters, Y‐tube tubes, and insect‐holding chamber. The Y‐tube was made of clear glass with the dimension of 14.0 × 3.0 cm (length × diameter). Components were connected with air tight silicon tubes

The repellency of 41 plant species to psyllids was determined using a Y‐tube olfactometer (Figure 1) in laboratory conditions. The design of the Y‐tube olfactometer followed that of Li et al. (2008). The Y‐tube olfactometer was made of an air pump, two air‐purifiers (activated carbon), two humidifiers, test and control chambers, two gas flow meters, two Y‐tube (clear glass), a light globe, and an insect‐holding chamber. The light globe was positioned above the two Y‐arms of the olfactometer to ensure that lighting to both arms was similar. The single arm of the Y‐tube was wrapped with black cloth. During the experiment, air was supplied by the air pump, filtered and humidified before passing through the test or control chambers. Air flow through the chambers to the two arms of the Y‐tube was regulated by an airflow meter, which was set at 400 ml/min for the duration of the experiment. The Y‐tube olfactometer was sterilized at 100°C for 30 min before the experiment. During assessment, the Y‐tube olfactometer was placed in horizontal position within a 1.0 × 0.6 × 0.6 m cardboard box. A fluorescent 1,600 lux daylight (5,000–6,500 K) globe, was mounted on the box to provide uniform lighting for the two Y‐tube arms.

The olfactometer was set up similarly to that of Zaka et al. (2010), but only leaves were used as volatile source materials. Leaves of the test and control plants were cleaned with distilled water, blot‐dried, and cut to small pieces of less than 2 mm; 5 g of leaves of test plants and 5 g of leaves of control plants were placed in the test chamber; 5 g of leaves of control plants only was placed in the control chamber.

The experiment was conducted in a laboratory at 25°C. At the start of each assessment, the freshly cut leaves were allowed to sit for 5 min to allow the plant volatiles to permeate throughout the Y‐tubes, then 40 healthy and active psyllids were released in the insect‐holding chamber. The number of psyllids entering the arms of the Y‐tube, passing the two‐third mark, and remaining for >30 s were recorded separately for both the test and control chamber arms. Each assessment was conducted for 20 min, starting from the time the psyllids were released in the holding chamber. All psyllids were removed after each assessment. The Y‐tube and the test and control chambers were cleaned, deodorized after each assessment by spraying with 75% alcohol then rinsing in distilled water three times before oven drying. For each test plant species, the assessment was repeated three times. Time laps between tests were approximately 15 min. Fresh psyllids were used in each assessment. We did not identify the sex of the collected psyllids. Each group of 40 individuals was randomly selected from a pool of psyllids collected a few hours before the experiment. We assumed that sex ratio between groups of psyllids (40) was similar and any sex‐related phenomenal response difference was therefore minimum.

In all, 4,920 psyllids were used in the assessment of the 41 test plant species.

### 2.4 Data analysis

An independent student's *t* test was used to determine the difference in number of psyllids entering the test and control arms of the Y‐tube for each plant species tested. ANOVA was used to examine the difference between test plant species using plant species as an independent factor and repellence as a dependent variable. The repellence was calculated as Wang et al. (2005):

$$\text{Repellence}\;\left(\%\right)=\left(T‐C\right)/\left(T+C\right)*100,$$

where T and C are the number of psyllids in the test and control Y‐tube arm, respectively.

The repellence ranged from 0 to 1. When T equaled C, that is, psyllid counts at the two Y‐tube arms are equal, repellence was 0, indicating the test plant species was not repellant. When C equaled 0, that is, no psyllids present in the test Y‐tube arm, repellence equaled 1, indicating the test plant species was strongly repellant. A dendrogram based on cluster analysis of the repellence was constructed to show grouping of the 41 test plant species on the basis of their relative repellence.

Student's *t* test and ANOVA were performed using SPSS 21.0 for Windows (SPSS Inc., Chicago, IL, USA). Cluster analysis was conducted using R version 3.6.3 (R‐Team, 2020) based on Euclidean distance and average linkage clustering.

## 3 RESULTS

The 41 plant species tested in this study were from 21 families (Table 1); 22 herbs, eight shrubs, and 11 trees. Twenty‐five of the species were collected from the local areas near Ganzhou. Fourteen were cultivars purchased from local nurseries of Ganzhou. Species in this category, including *Allium cepa*, *Capsicum annuum*, and *Nicotiana tabacum*, are highly aromatic and widely cultivated species of economic importance. The other two species, *Camptotheca acuminata* and *Ginkgo biloba* were collected from the university campus in Wuhan.

Repellency to ACP showed marked variation between the 41 plant species tested (ANOVA, *df* = 40,82, *F* = 13.2, *p* < .01; Table 2). Repellency was strongest in *Mimosa bimucronata* and weakest in *Crepidiastrum sonchifolium* (repellency of 76% and 8.3%, respectively). Independent *t* test showed that for 10 species, the mean number of psyllid entering Y‐tube arm connected to the treatment plant was significantly lower than that connected to the control plant arm (*Citrus reticulata* cv. Murcott) (*t* test, *p* < .05). With five of the test plant species, in order of repellency, *M. bimucronata*, *C. annuum*, *Lantana camara*, *Gynura bicolor*, and *C. acuminata*, the mean number of psyllids was five or less, which represented only one‐eighth of the 40 psyllids used in the assessments. Using the number of visits to the control arm as the base (100%), visits to the testing arm were reduced by 86% to 75%. These differences were statistically highly significant (*t* test, *p* < .01). For the remaining 31 test plant species, the differences in mean psyllid number between the test arm and the control arm were not statistically significant (*t* test, *p* ≥ .05), suggesting these species had no or minimum repelling effects on ACP.

**TABLE 2:** Psyllid counts (mean ± *SE*) and repellency (%) of 41 plant species sorted in order of decreasing repellence

Test plant species	Psyllid number		Repellence (%)	*t* test Treatment versus control
	Test	Control		
*Mimosa bimucronata*	4.33 ± 0.47	31.33 ± 1.70	75.72	**
*Capsicum annuum*	4.67 ± 1.25	29.33 ± 3.68	72.53	**
*Lantana camara*	5.33 ± 1.25	23.00 ± 1.63	62.37	**
*Gynura bicolor*	4.33 ± 0.94	18.67 ± 2.36	62.35	**
*Camptotheca acuminata*	4.33 ± 1.25	17.00 ± 1.63	59.40	**
*Nicotiana tabacum*	5.67 ± 0.94	17.67 ± 1.25	51.41	*
*Psidium guajava*	5.67 ± 0.47	16.67 ± 1.25	49.24	*
*Tuberosum rottl*	8.33 ± 1.25	21.00 ± 2.16	43.20	*
*Cinnamomum burmanni*	6.67 ± 2.05	15.67 ± 2.49	40.29	n.s.
*Bidens tripartita*	9.33 ± 1.25	21.33 ± 1.7	39.14	*
*Melia azedarach*	7.33 ± 0.40	16.67 ± 2.05	38.92	n.s.
*Mentha haplocalyx*	8.00 ± 0.82	17.67 ± 0.94	37.67	n.s.
*Cinnamomum camphora*	7.00 ± 0.82	14.67 ± 3.68	35.39	n.s.
*Celtis sinensis*	9.67 ± 1.70	17.67 ± 3.4	29.26	n.s.
*Pittosporum tobira*	7.33 ± 0.47	13.00 ± 1.63	27.89	n.s.
*Allium cepa*	9.67 ± 1.25	16.00 ± 0.82	24.66	n.s.
*Ocimum basilicum*	10.00 ± 0.82	15.67 ± 1.7	22.09	n.s.
*Artemisia lavandulaefolia*	10.67 ± 1.70	16.33 ± 1.25	20.96	n.s.
*Rubus parvifolius*	7.67 ± 0.47	11.67 ± 1.25	20.68	n.s.
*Pinus massoniana*	10.33 ± 0.94	15.67 ± 1.25	20.54	n.s.
*Ricinus communis*	8.67 ± 1.25	15.67 ± 0.94	20.38	n.s.
*Imperata cylindrica*	9.33 ± 0.47	14.00 ± 1.41	20.02	n.s.
*Corchorus acutangulus*	9.33 ± 1.25	14.00 ± 1.63	20.01	n.s.
*Ginkgo biloba*	9.33 ± 1.25	13.67 ± 1.25	18.87	n.s.
*Tagetes erecta*	11.67 ± 2.49	17.00 ± 0.82	18.59	n.s.
*Polygonum perfoliatum*	9.67 ± 0.47	13.67 ± 0.47	17.14	n.s.
*Paliurus ramosissimus*	10.67 ± 1.25	15.00 ± 0.82	16.87	n.s.
*Zanthoxylum bungeanum*	11.00 ± 0.82	15.33 ± 1.25	16.45	n.s.
*Allium sativum*	9.33 ± 1.70	13.00 ± 2.45	16.44	n.s.
*Bidens pilosa*	12.00 ± 1.63	16.67 ± 1.25	16.29	n.s.
*Allium fistulosum*	10.33 ± 1.25	14.33 ± 1.25	16.22	n.s.
*Cosmos bipinnatu*	12.00 ± 0.82	16.33 ± 1.25	15.28	n.s.
*Osmanthus fragrans*	10.00 ± 0.82	13.33 ± 1.25	14.27	n.s.
*Rhodomyrtus tomentosa*	10.33 ± 1.25	13.67 ± 1.25	13.92	n.s.
*Xanthium sibiricum*	10.67 ± 1.25	14.00 ± 0.82	13.50	n.s.
*Amorpha fruticosa*	9.67 ± 1.25	12.67 ± 1.25	13.43	n.s.
*Cinnamomum japonicum*	12.33 ± 0.94	16.00 ± 0.82	12.95	n.s.
*Sapium sebiferum*	10.33 ± 1.2a	13.33 ± 0.47	12.68	n.s.
*Coriandrum sativum*	12.33 ± 0.94	15.33 ± 0.47	10.85	n.s.
*Abelmoschus esculentus*	15.67 ± 1.89	19.00 ± 1.63	9.60	n.s.
*Crepidiastrum sonchifolium*	11.00 ± 2.16	13.00 ± 0.82	8.33	n.s.

Note

*p < .05; **p < .01; n.s., not significant, *p* ≥ .05. For each species tested, three assessments were conducted with 40 psyllids used in each assessment. *Citrus reticulata* cv. Murcott was used as control plant.

**FIGURE 2:**
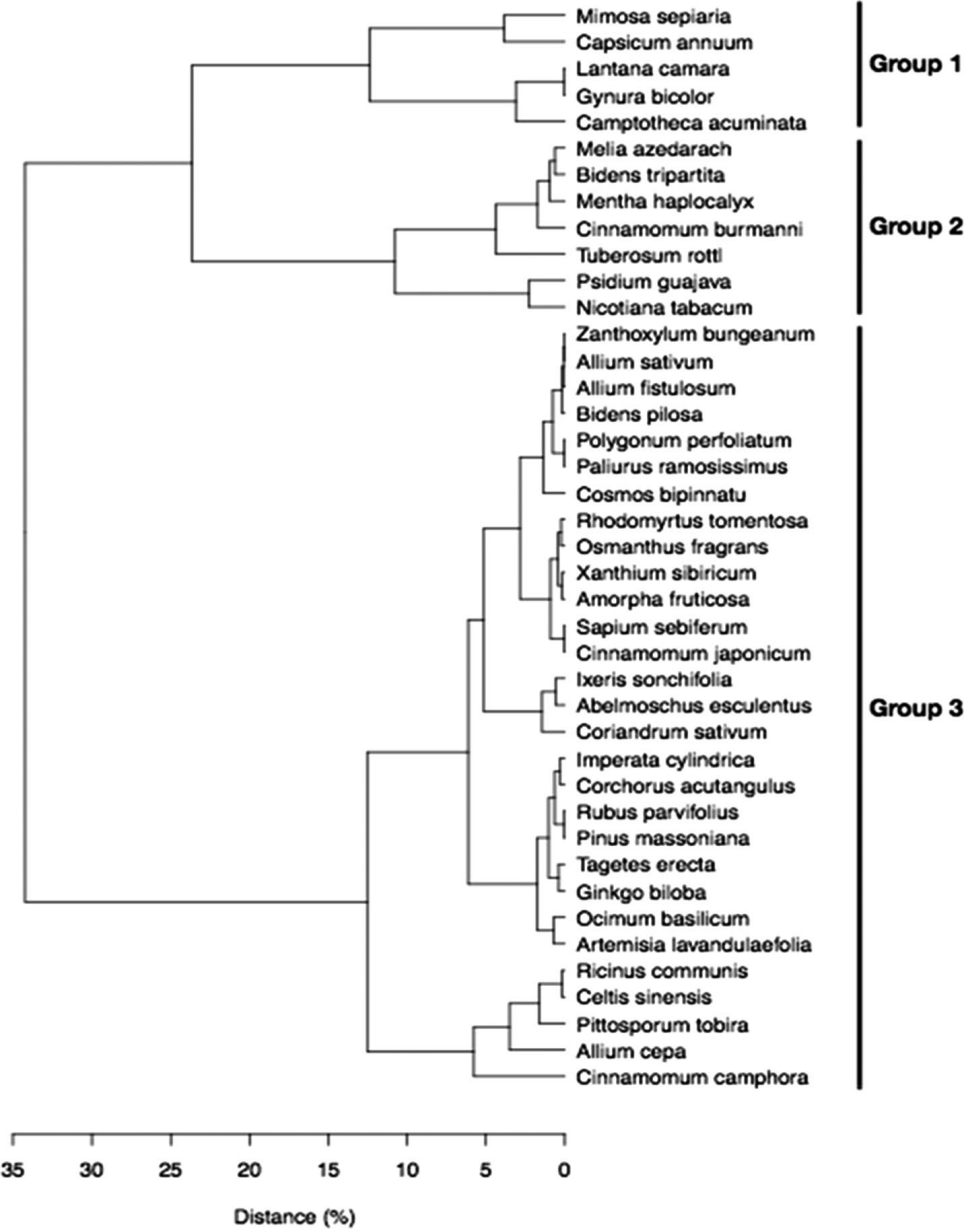
Dendrogram showing grouping of 41 aromatic plant species based on their repellency (%) to Asian citrus psyllids (*Diaphorina citri*). Dendrogram constructed in R version 3.6.3 (R‐Team, 2020) based on Euclidean distance and average linkage clustering. Three groups with >24% dissimilarity were clearly identified

A dendrogram grouping the 41 test plant species based on cluster analysis of their repellency to ACP is given in Figure 2. Three groups with >24% dissimilarity were clearly identified. The first group consisted of the five species that had the highest repellency (59%–76%) and lowest mean number of psyllids entering the testing arm of treatment plants (Table 2). The second group consisted of seven species that had repellency of 38%–51%, the mean number of psyllids were also significantly lower on test plants than on the controls (*t* test, *p* < .05), with the exceptions of *Cinnamomum burmannii* and *Mentha haplocalyx*, which have repellency of 40% and 39%, respectively. The third group consisted of the remaining 29 plant species. The repellency of species from this group was generally low (<40%) and the difference in mean psyllid number on the teat plants was not significantly different from the control (*t* test, *p* ≥ .05).

## 4 DISCUSSION

There is no known treatment for *Ca*. L. asiaticus infected plants. Unlike other diseases, in HLB‐endemic regions, disease prevention is more important than treatment (Wang, 2019). Efficient control of the vector, ACP, is key to successful HLB prevention. In Brazil, controlling ACP has been effective in reducing HLB incidence to a level that is economically sustainable (Bassanezi et al., 2013). In citrus‐producing areas of China, orchards remain productive and profitable with an infection incidence below 30% (Wang, 2019). However, the actual *Ca*. L. asiaticus incidence may be much higher due to the latent period (up to 5 years) between *Ca*. L. asiaticus infection and symptom expression. Thus, in these regions, the aim is to keep the number of *Ca*. L. asiaticus infected trees below the 30% threshold (symptom based).

Using aromatic plants to reduce the abundance of ACP so that *Ca*. L. asiaticus incidence falls below this threshold is a promising management option. The practice of using plant volatiles to repel insects has been used for pest management in horticulture production such as potato (Berberich, 1988), pears (Tang et al., 2013) as well as in citrus (Fancelli et al., 2018; Yan et al., 2015). Incidentally, the repelling effects of volatiles of guava on *D. citri* were discovered by accident rather than by planned research. In 2004 in tropical Vietnam, citrus growers used guava as an intercropping plant to increase short‐term cash flow as it fruits within 1 year compared to 3 or more years for citrus trees (Ichinose et al., 2012). They reported a lower incidence of HLB in these interplanted orchards. The efficacy of guava interplanting in citrus HLB was limited to 1 year. Further experiments demonstrated that interplanting of guava with citrus trees can effectively reduce the density of psyllids, leading to the lower level of HLB infected plants (Ichinose et al., 2012). These findings have been confirmed by several other studies that show volatiles of guava leaves are an effective repellent for psyllids and contribute to the reduced HLB incidence (Barman et al., 2016; Gottwald et al., 2014; Onagbola et al., 2011; Silva et al., 2016; Zaka et al., 2010).

In our study, the five plants in the group with the highest repellency of ACP (Figure 2) could be useful for interplanting or as a landscape barrier to deter ACP from citrus orchards. Under laboratory conditions, volatiles of these species were able to reduce ACP numbers by 75%–87% (Table 2). Among these species, *Camptotheca acuminate* is a deciduous tree that can grow to 20 m. Both *L*. c*amara* and *M. bimucronata* are shrubs that can grow to 2 and 6 m, respectively. These three species could be planted around the perimeter of citrus orchards as landscape barriers to ACP. Landscape barriers, consisting of trees or shrubs, can serve as windbreaks and have been reported to reduce the abundance of *D. citri* in orchards and protect them from HLB (Martini et al., 2015). *Gynura bicolor* and *L. camara* are both known for their use as medicinal plants. *Gynura bicolor* is a herb and has been used for treating diseases such as dysmenorrhea, dysentery, and ulcers. *Lantana camara* has been used for treating diseases such fever, mumps, and tuberculosis. The fifth species, *C. annuum* commonly grown for its fruit. These species all have higher repellency rate than *Psidium guajava* (73%–59% vs. 49%) (Table 2), the species that has previously been reported to be repellent to ACP and capable of reducing the level of HLB (Ichinose et al., 2012; Silva et al., 2016), suggesting these species offer great potential for managing HLB. *P. guajava* is a tropical species, hence its use as a repellent plant is restricted to the tropical regions. In contrast, *C. annuum*, *G. bicolor*, and *L. camara* are cultivated worldwide, thus are potentially suitable for planting in all citrus‐growing regions. Both *C. annuum* and *G. bicolor* are annuals grows in spring‐autumn, coinciding with ACP active season, thus making them ideal candidates for HLB management. Both *C. annuum* and *G. bicolor* can be used for intercropping in the orchards. Apart from the repelling effects on ACP, these plants themselves will be of economic value to the growers, thereby potentially improving the economics and sustainability of the citrus industry.

Our repellency study was based on leaves only. While leaves of all tested species showed the strongest volatile emitting characteristics among all plant parts (shoots, stems, flowers, and fruits), the overall repellency of given species to ACP may differ between leaves and the plant as whole. To ascertain the repellency of the five species showed the highest repellency to ACP in the field, further field studies are needed. These studies should take into consideration: (a) interplanting species (single vs. multiple); (b) planting layout (row spacing, planting density; interplanting vs. perimeter planting); (c) interaction with citrus production (effects on pollinators, natural enemies of ACP and citrus); and (d) economics (costs vs. income).

Compared to other options, such as pruning the diseased plants and chemical control of ACP (Bove, 2006; Wang, 2019), an integrated pest management approach using volatile plants offers the best option for HLB management. The integrated pest management option is more economical, as it does not require the amount of labor input as pruning does. In addition, the extra income derived from products of interplanting crops will provide added value for the citrus growers. Integrated pest management will also be environmentally friendly. Using insecticides to control ACP is increasingly been viewed as a hazard both to the citrus workers and to the environment (Beloti et al., 2015; Wang, 2019).

## 5 CONCLUSIONS

In conclusion, our results supported the hypothesis that plant volatiles can effectively repel ACP, the insect vector of the bacterium associated with HLB. Volatiles of five plant species were most effective in repelling ACP, with a repellency as high as 76%. Among these, the tree species, *C. acuminate*, *a*nd the two shrub species, *L. camara* and *M. bimucronata* could be planted around the perimeter of citrus orchards and used as a landscape barrier to the vector. The two herb species, *C. annuum* and *G. bicolor*, could be intercropped with citrus trees to repel ACP. Although further field evaluation of various interplanting regimes and landscape barriers are needed to assess their effectiveness, our results showed that these aromatic species, being highly repellent to ACP, offer great potential as more cost‐effective and environmentally sustainable alternatives to the current methods of managing HLB.

## CONFLICT OF INTEREST

There is no conflict of interest/competing interest.

## AUTHOR CONTRIBUTIONS

**Zhaogui Yan:** Data curation (equal); formal analysis (lead); writing – review & editing (lead). **Qun Zhang:** Data curation (equal); formal analysis (equal); investigation (equal); methodology (equal); writing – review & editing (equal). **Yongrong Xu:** Conceptualization (lead); funding acquisition (lead); writing – review & editing (equal). **Nan Zhang:** Data curation (supporting); investigation (equal); writing – review & editing (supporting). **Wan Li:** Data curation (equal); formal analysis (equal); investigation (equal). **Cuiying Chang:** Data curation (equal); formal analysis (equal); investigation (equal). **Yan Xiang:** Formal analysis (equal); investigation (equal); writing – review & editing (equal). **Wei He:** Formal analysis (equal); writing – review & editing (equal). **Tengyu Jiang:** Formal analysis (equal). **Changxiu Xia:** Investigation (equal); methodology (equal). **Jie Luo:** Formal analysis (equal); methodology (equal).

## Data Availability

Data on experiment materials and repellency measurements were archived in Dryad. The data access DOI number is https://doi.org/10.5061/dryad.m0cfxpp1v.

## References

[ece36876-bib-0001] Barman, J. C. , Campbell, S. A. , & Zeng, X. N. (2016). Exposure to guava affects citrus olfactory cues and attractiveness to *Diaphorina citri* (Hemiptera: Psyllidae). Environmental Entomology, 45(3), 694–699. 10.1093/ee/nvw010 27247354

[ece36876-bib-0002] Bassanezi, R. B. , Montesino, L. H. , Gimenes‐Fernandes, N. , Yamamoto, P. T. , Gottwald, T. R. , Amorim, L. , & Bergamin, A. (2013). Efficacy of area‐wide inoculum reduction and vector control on temporal progress of huanglongbing in young sweet orange plantings. Plant Disease, 97(6), 789–796. 10.1094/Pdis-03-12-0314-Re 30722592

[ece36876-bib-0003] Bassanezi, R. B. , Montesino, L. H. , & Stuchi, E. S. (2009). Effects of huanglongbing on fruit quality of sweet orange cultivars in Brazil. European Journal of Plant Pathology, 125(4), 565–572. 10.1007/s10658-009-9506-3

[ece36876-bib-0004] Beloti, V. H. , Alves, G. R. , Araujo, D. F. D. , Picoli, M. M. , Moral, R. D. , Demetrio, C. G. B. , & Yamamoto, P. T. (2015). Lethal and sublethal effects of insecticides used on citrus, on the ectoparasitoid *Tamarixia radiata* . PLoS One, 10(7), e0132128 10.1371/journal.pone.0132128 26132327 PMC4488444

[ece36876-bib-0005] Berberich, S. (1988). Potato plants make their own insect repellent. Agricultural Research, 36(2), 15.

[ece36876-bib-0006] Bove, J. M. (2006). Huanglongbing: A destructive, newly‐emerging, century‐old disease of citrus. Journal of Plant Pathology, 88(1), 7–37.

[ece36876-bib-0007] Diaz, J. H. (2016). Chemical and plant‐based insect repellents: Efficacy, safety, and toxicity. Wilderness & Environmental Medicine, 27(1), 153–163. 10.1016/j.wem.2015.11.007 26827259

[ece36876-bib-0008] Fancelli, M. , Borges, M. , Laumann, R. A. , Pickett, J. A. , Birkett, M. A. , & Blassioli‐Moraes, M. C. (2018). Attractiveness of host plant volatile extracts to the Asian citrus psyllid, *Diaphorina citri*, is reduced by terpenoids from the non‐host cashew. Journal of Chemical Ecology, 44(4), 397–405. 10.1007/s10886-018-0937-1 29500752 PMC5899996

[ece36876-bib-0009] Gottwald, T. R. (2010). Current epidemiological understanding of citrus huanglongbing. Annual Review of Phytopathology, 48(48), 119–139. 10.1146/annurev-phyto-073009-114418 20415578

[ece36876-bib-0010] Gottwald, T. R. , Hall, D. G. , Kriss, A. B. , Salinas, E. J. , Parker, P. E. , Beattie, G. A. C. , & Nguyen, M. C. (2014). Orchard and nursery dynamics of the effect of interplanting citrus with guava for huanglongbing, vector, and disease management. Crop Protection, 64, 93–103. 10.1016/j.cropro.2014.06.009

[ece36876-bib-0011] Halbert, S. E. , & Manjunath, K. L. (2004). Asian citrus psyllids (Sternorrhyncha : Psyllidae) and greening disease of citrus: A literature review and assessment of risk in Florida. Florida Entomologist, 87(3), 330–353. 10.1653/0015-4040(2004)087[0330:Acpspa]2.0.Co;2

[ece36876-bib-0012] Hall, D. G. , Gottwald, T. R. , Stover, E. , & Beattie, G. A. C. (2013). Evaluation of management programs for protecting young citrus plantings from huanglongbing. HortScience, 48(3), 330–337. 10.21273/Hortsci.48.3.330

[ece36876-bib-0013] Hill, N. , Lenglet, A. , Arnez, A. M. , & Carneiro, I. (2007). Plant based insect repellent and insecticide treated bed nets to protect against malaria in areas of early evening biting vectors: Double blind randomised placebo controlled clinical trial in the Bolivian Amazon. British Medical Journal, 335(7628), 1023–1025. 10.1136/bmj.39356.574641.55 17940319 PMC2078668

[ece36876-bib-0014] Ichinose, K. , Hoa, N. V. , Bang, D. V. , Tuan, D. H. , & Dien, L. Q. (2012). Limited efficacy of guava interplanting on citrus greening disease: Effectiveness of protection against disease invasion breaks down after one year. Crop Protection, 34, 119–126. 10.1016/j.cropro.2011.11.023

[ece36876-bib-0015] Ichinose, K. , Miyazi, K. , Matsuhira, K. , Yasuda, K. , Sadoyama, Y. , Do, H. T. , & Doan, V. B. (2010). Unreliable pesticide control of the vector psyllid *Diaphorina citri* (Hemiptera: Psyllidae) for the reduction of microorganism disease transmission. Journal of Environmental Science and Health Part B‐Pesticides Food Contaminants and Agricultural Wastes, 45(5), 466–472. 10.1080/03601231003800263 20512737

[ece36876-bib-0016] Jimenez, M. I. G. , & Poveda, K. (2009). Synergistic effects of repellents and attractants in potato tuber moth control. Basic and Applied Ecology, 10(8), 763–769. 10.1016/j.baae.2009.06.009

[ece36876-bib-0017] Karunamoorthi, K. , Ilango, K. , & Endale, A. (2009). Ethnobotanical survey of knowledge and usage custom of traditional insect/mosquito repellent plants among the Ethiopian Oromo ethnic group. Journal of Ethnopharmacology, 125(2), 224–229. 10.1016/j.jep.2009.07.008 19607902

[ece36876-bib-0018] Li, J. , Wang, M. , Zhang, Z. , & Chen, J. (2008). The behavioral response of adult *Cercosporas* to plant odor. Forest Sciences, 6, 168–170 (in Chinese).

[ece36876-bib-0019] Martini, X. , Pelz‐Stelinski, K. S. , & Stelinski, L. L. (2015). Absence of windbreaks and replanting citrus in solid sets increase density of Asian citrus psyllid populations. Agriculture Ecosystems & Environment, 212, 168–174. 10.1016/j.agee.2015.06.027

[ece36876-bib-0020] Moore, S. J. , Lenglet, A. , & Hill, N. (2002). Field evaluation of three plant‐based insect repellents against malaria vectors in Vaca Diez province, the Bolivian Amazon. Journal of the American Mosquito Control Association, 18(2), 107–110. 12083351

[ece36876-bib-0021] Naeem, A. , Freed, S. , Jin, F. L. , Akmal, M. , & Mehmood, M. (2016). Monitoring of insecticide resistance in *Diaphorina citri* Kuwayama (Hemiptera: Psyllidae) from citrus groves of Punjab, Pakistan. Crop Protection, 86, 62–68. 10.1016/j.cropro.2016.04.010

[ece36876-bib-0022] Onagbola, E. O. , Rouseff, R. L. , Smoot, J. M. , & Stelinski, L. L. (2011). Guava leaf volatiles and dimethyl disulphide inhibit response of *Diaphorina citri* Kuwayama to host plant volatiles. Journal of Applied Entomology, 135(6), 404–414. 10.1111/j.1439-0418.2010.01565.x

[ece36876-bib-0023] Plotto, A. , Baldwin, E. , McCollum, G. , Manthey, J. , Narciso, J. , & Irey, M. (2010). Effect of *Liberibacter* infection (huanglongbing or “greening” disease) of citrus on orange juice flavor quality by sensory evaluation. Journal of Food Science, 75(4), S220–S230. 10.1111/j.1750-3841.2010.01580.x 20546425

[ece36876-bib-0024] Qin, L. H. (2018). Research progress on occurrence and epidemic of citrus yellow dragon disease. Modern Agriculture Perspective, 22, 110–116 (in Chinese).

[ece36876-bib-0025] Rouse, R. E. , Ozores‐Hampton, M. , Roka, F. M. , & Roberts, P. (2017). Rehabilitation of huanglongbing‐affected citrus trees using severe pruning and enhanced foliar nutritional treatments. HortScience, 52(7), 972–978. 10.21273/Hortsci11105-16

[ece36876-bib-0026] R‐Team (2020). R: A language and environment for statistical computing. R Foundation for Statistical Computing Retrieved from https://www.R-project.org

[ece36876-bib-0027] Silva, J. A. A. , Hall, D. G. , Gottwald, T. R. , Andrade, M. S. , Maldonado, W. , Alessandro, R. T. , Lapointe, S. L. , Andrade, E. C. , & Machado, M. A. (2016). Repellency of selected *Psidium guajava* cultivars to the Asian citrus psyllid, *Diaphorina citri* . Crop Protection, 84, 14–20. 10.1016/j.cropro.2016.02.006

[ece36876-bib-0028] Tang, G. B. , Song, B. Z. , Zhao, L. L. , Sang, X. S. , Wan, H. H. , Zhang, J. , & Yao, Y. C. (2013). Repellent and attractive effects of herbs on insects in pear orchards intercropped with aromatic plants. Agroforestry Systems, 87(2), 273–285. 10.1007/s10457-012-9544-2

[ece36876-bib-0029] US Department of Agriculture (2018). Florida citrus statistics 2016–2017. US Department of Agriculture, National Agricultural Statistics Service.

[ece36876-bib-0030] Vashisth, T. , & Livingston, T. (2019). Assessment of pruning and controlled‐release fertilizer to rejuvenate Huanglongbing‐affected sweet orange. Horttechnology, 29(6), 933–940. 10.21273/Horttech04382-19

[ece36876-bib-0031] Wang, J. , Li, Y. , & Lei, Z. (2005). Repellency and fumigation activity of *Artemisia argyi* essential oil to housefly. Entomology Studies, 42, 51–53 (in Chinese).

[ece36876-bib-0032] Wang, N. (2019). The citrus huanglongbing crisis and potential solutions. Molecular Plant, 12(5), 607–609. 10.1016/j.molp.2019.03.008 30947021

[ece36876-bib-0033] Yan, H. X. , Zeng, J. W. , & Zhong, G. Y. (2015). The push‐pull strategy for citrus psyllid control. Pest Management Science, 71(7), 893–896. 10.1002/ps.3915 25256398

[ece36876-bib-0034] Yang, K. , Guo, S. S. , Zhang, W. J. , Wang, C. F. , Han, J. , Geng, Z. F. , & Deng, Z. W. (2015). Repellent activity of *Glycosmis* plant extracts against two stored product insects. Boletin Latinoamericano Y Del Caribe De Plantas Medicinales Y Aromaticas, 14(6), 462–469.

[ece36876-bib-0035] Zaka, S. M. , Zeng, X. N. , Holford, P. , & Beattie, G. A. C. (2010). Repellent effect of guava leaf volatiles on settlement of adults of citrus psylla, *Diaphorina citri* Kuwayama, on citrus. Insect Science, 17(1), 39–45. 10.1111/j.1744-7917.2009.01271.x

[ece36876-bib-0036] Zheng, Z. , Chen, J. C. , & Deng, X. L. (2018). Historical perspectives, management, and current research of citrus HLB in Guangdong province of China, where the disease has been endemic for over a hundred years. Phytopathology, 108(11), 1224–1236. 10.1094/Phyto-07-18-0255-Ia 30156499

